# Prevalence of Posttraumatic Stress Disorder among Firefighters in Iran: A Systematic Review and Meta-Analysis

**DOI:** 10.18502/ijps.v15i4.4301

**Published:** 2020-10

**Authors:** Ali Sahebi, Kosar Yousefi, Siamak Moayedi, Najaf Golitaleb, Ali Esmaeili Vardanjani, Mohamad Golitaleb

**Affiliations:** 1 Department of Health in Disasters and Emergencies, School of Public Health and Safety, Shahid Beheshti University of Medical Sciences, Tehran, Iran.; 2 Clinical Research Development Unit, Shahid Mostafa Khomeini Hospital, Ilam University of Medical Sciences, Ilam, Iran.; 3 Department of Emergency Medicine, University of Maryland School of Medicine, Baltimore, United States.; 4 Social Determinants of Health Research Center, Lorestan University of Medical Sciences, Khorramabad, Iran.; 5 Department of Critical Care Nursing and Management, School of Nursing and Midwifery, Tehran University of Medical Sciences, Tehran, Iran.; 6 School of Nursing and Midwifery, Hamadan University of Medical Sciences, Hamadan, Iran.; 7 Department of Critical Care Nursing, School of Nursing, Arak University of Medical Sciences, Arak, Iran.

**Keywords:** *First Responder*, *Firefighters*, *Mental Health*, *Posttraumatic Stress Disorder (PTSD)*

## Abstract

**Objective:** Firefighters are exposed to many different chemicals and physical hazards and experience a significant number of accidents and injuries. They are exposed to high-risk of posttraumatic stress disorder (PTSD).

The purpose of this study was to determine the prevalence of PTSD among Iranian firefighters.

**Method** **:** This was a systematic review and meta-analysis. Valid Persian and English keywords were searched in data resources, including SID, Magiran, Irandoc, Google Scholar, PubMed, Scopus, and Web of Science to retrieve articles on the prevalence of PTSD among Iranian firefighters. The STROBE checklist was used to assess the quality of the articles. Heterogeneity among the studies was assessed by I^2^ index. The data were analyzed using Stata14 software.

**Results: **Of the 316 articles selected in the initial search, 3 articles the inclusion criteria and were used for the meta-analysis. A total of 274 firefighters were studied. The prevalence of PTSD was 23.17% among Iranian firefighters (I^2^ = 92.4%, 95% CI = 6.71-39.62, p < 0.001).

**Conclusion: **The prevalence of PTSD is among Iranian firefighters relatively high and has been on the rise over the recent years.

Considering the nature of duties and emergency services, firefighters experience a significant number of accidents and face various physical and chemical hazards, including extreme heat, toxic compounds, and loud noises (1, 2). Therefore, compared with other communities, firefighters are more exposed to various injuries and stresses exposing them to depression, Posttraumatic Stress Disorder (PTSD), and other problems (3). Firefighters are among high-risk groups to develop psychological problems. Thus, it is critical to closely monitor them for such diseases to boost their functionality and reduce human error rates (4). In a study by Chen et al (5) the relationship between quality of life and PTSD or major depression was examined among Firefighters. This study revealed a high rate of PTSD, which negatively affected the quality of life in these individuals. In another study by Heinrichs et al (6), the risk factors of PTSD was monitored in firefighters for 2 years. They found significant increases in PTSD, depression, anxiety, and other mental illnesses in highly-aggressive participants and those who had low levels of self-effectiveness.

PTSD is a complex physical, cognitive, emotional, behavioral, and mental health disorder characterized by disturbing thoughts, feeling of hopelessness, tendency to avoid trauma memories, anxiety, and ultimately social, occupational, and communicational disturbances (7). 

PTSD usually occurs following a life-threatening event (7, 8). The risk of PTSD is high in individuals such as hospital workers, military staff, police officers, and firefighters who are exposed to severe psychological traumas and occupational distresses (9, 10). PTSD negatively affects the quality of life and social relationships and increases the risk of suicide among firefighters (11). The prevalence of suicidal ideation and suicide attempt among firefighters have been documented as 44% and 25%, respectively (11, 12). The risk of PTSD among firefighters varies in the literature from 6.5% to 50%, which is significantly higher than that in the general population (13-16). PTSD causes such symptoms as fatigue, loss of concentration, inactivity, sleep disturbance, social apathy, avoidance behavior, workplace absenteeism, and problems in social performance and relationships (17, 18). Personal characteristics, longer work experience, increased number of distressing missions, and exposure to severe traumatic events are among the factors contributing to the risk of PTSD among firefighters (19, 20). Given the high prevalence of PTSD among firefighters and the impact of this condition on quality of life, risk of suicide and social communications, it is imperative to promptly identify individuals at risk of PTSD. 

Because PTSD negatively affects the mental status, performance, and social life of people, determining the prevalence of this disorder is important to control its progression (21). In addition, understanding the health impacts of PTSD can be helpful in implementing preventive measures, improving employees’ mental function, increasing productivity and satisfaction rates, reducing human-born errors, and finally upgrading the quality of services. Considering that firefighters are vulnerable to PTSD and that limited studies have been conducted on the prevalence of this disorder among Iranian firefighters, we aimed to collect and analyze these limited studies to estimate an overall prevalence of PTSD in Iran, particularly in Iranian firefighters.

## Materials and Methods

This was a systematic review and meta-analysis, based on the guidelines of the preferred reporting items for systematic reviews and meta-analyses (PRISMA) (22). According to this guideline, selecting and qualifying studies and extracting data were independently performed by 2 authors; and in case of any disagreement between them, a decision was made through group discussion. 

***Search Strategy***

The literature search was conducted in the data resources of Irandoc, Google Scholar, SID, Magiran, Scopus, Science Direct, PubMed, and Web of Science. The keywords in English and Persian included posttraumatic stress disorder, posttraumatic neuroses, Post Traumatic Stress Disorders, chronic post-traumatic stress disorder, delayed-onset post-traumatic stress disorder, acute post-traumatic stress disorder, PTSD, firefighter, fire fighter, fire and rescue personnel and Iran. Search operators of (AND) and (OR) were used to perform combinational searches. The articles published up to June 2019 were selected. 

***Inclusion Criteria***

Studies either in English or Persian that determined the prevalence of PTSD in Iranian firefighters using valid scientific tools were included.

***Exclusion Criteria***

Studies not reporting the prevalence of PTSD and full-text unavailability were considered as exclusion criteria.

***Selection Study***

After an extensive search on the mentioned resources, 316 studies were initially inserted into the Endnote. After removing duplicate studies, titles and abstracts of 265 articles were assessed. In the next step, 2 researchers independently reviewed full-texts of 14 related studies in detail. Finally, 3 studies were selected for qualitative evaluation.

***Quality Assessment***

Quality assessment was performed using the standard 22-item STROBE checklist (23). The minimum and maximum scores based on this checklist were 0 and 44, respectively. Studies that attained at least 16 scores were included in the meta-analysis.

***Data Extraction***

The data from all the included articles were recorded into a preprepared checklist. The data included first authors’ name, location of study, year of publication, type of study, sample size, mean age of participants, tool used in the study, and the prevalence of PTSD among firefighting personnel. 

***Statistical Analysis***

As the prevalence of PTSD and the sample size were characterized for each study. The I^2^ index was used to assess the heterogeneity of the studies. Due to the high and significant heterogeneity among the studies (ie, I^2^ high heterogeneity index), the meta-analysis was conducted using the random effects model. The I^2^ values of <25%, 25-75%, and >75% represent low, moderate, and high heterogeneity, respectively (24). Meta-regression analysis was used to examine the association between the prevalence of PTSD and the year of study. The Egger test was used to assess publication bias. The data were analyzed using STATA software (version 14). 

## Results

**Figure 1:**
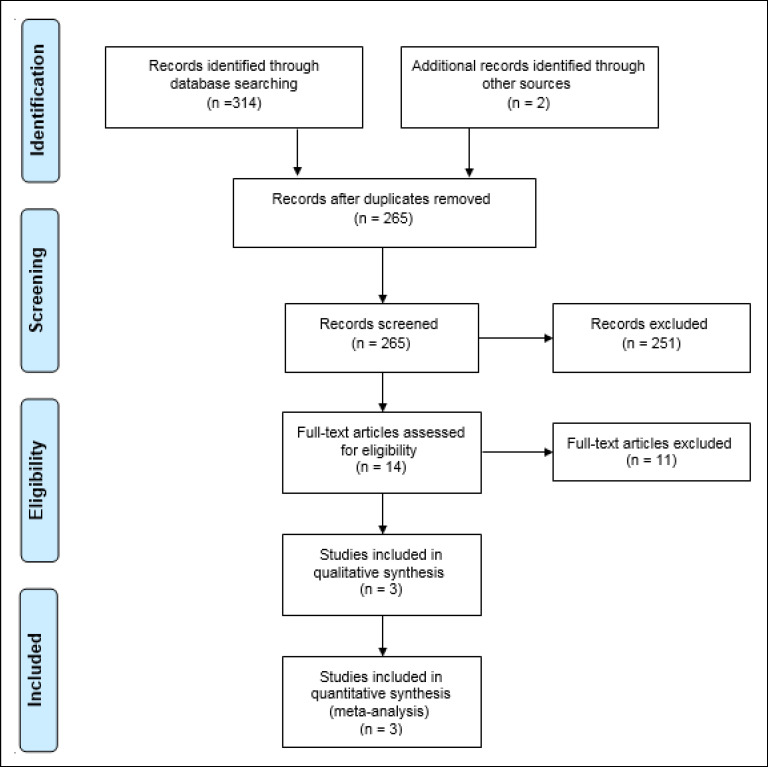
Flowchart of the Selection of Studies Based on PRISMA

**Figure 2:**
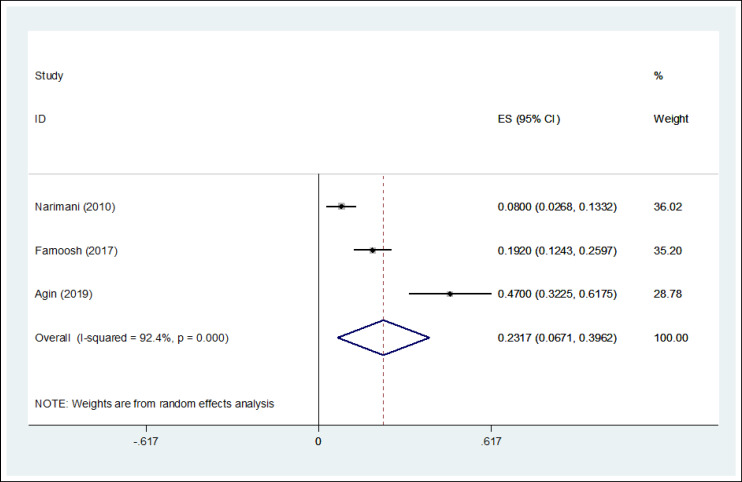
The Forest Plot of Overall and Individual PTSD Prevalence in the Included Studies with 95% Confidence Interval

**Figure 3:**
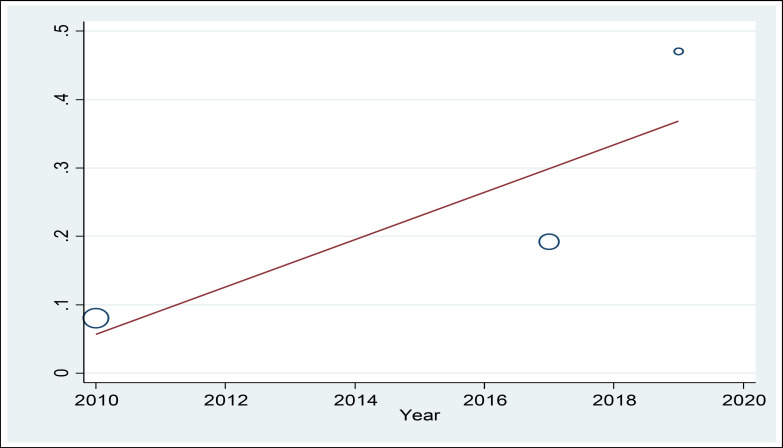
The Meta-Regression Analysis of the Studies Based on the Year of Publication

**Figure 4:**
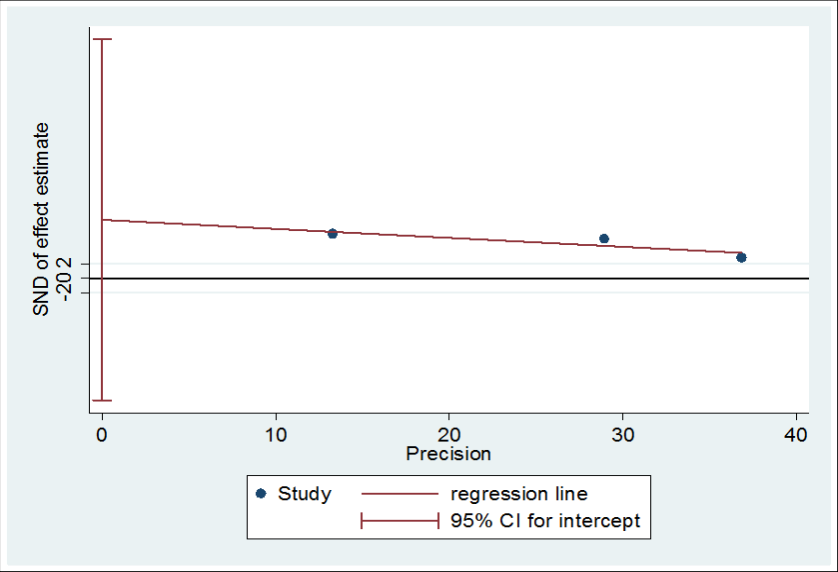
Publication Bias Based on Egger Test

In this systematic review, 316 studies related to the topic were identified during the initial search, of which 313 were excluded because they failed to meet the inclusion criteria. Finally, 3 studies published between 2010 and 2019 were evaluated using the STROBE checklist and included in the meta-analysis process. Figure 1 shows the process of study selection. The total number of participants was 274. The selection of participants was based on simple random sampling in most of the studies, and all of the studies had a cross sectional design. The mean age of the participants was 35.21 ± 5.24 years. Table 1 shows the general features and the data extracted from each study. According to the statistical analysis, the overall prevalence of PTSD was 23.17% (95% CI = 6.71- 39.62, I^2^ = 92.4%, p < 0.001) (Figure 2). The results of meta-regression showed that the prevalence of PTSD based on the year of study indicated an increasing trend (Figure 3). The results of Egger test showed that the effect of publication bias was not significant (p = 0.151) (Figure 4). 

**Table 1:** The Specifications of Studies Included in the Meta-Analysis

**First Author**	**Place**	**Year**	**Type of Study**	**Sample Size**	**Average ** **Age(SD)**	**Instrument**	**Prevalence of PTSD**
Agin(25)	Tehran	2019	cross-sectional	44	32.8(6.6)	Watson	47%
Nariman(26)	Uremia	2010	cross-sectional	100	41.23(13.73)	Mississippi	8%
Farnoosh (27)	Tehran	2017	cross-sectional	130	31.6(3.67)	Mississippi	19.2%

## Discussion

PTSD is one of the most common, chronic, and debilitating psychiatric disorders. According to the Diagnostic and Statistical Manual of Mental Disorders, 5th Edition: DSM-5, it is categorized as “a disorder related to trauma and stress developing upon exposition to a traumatic event.” The disease is characterized by 4 cluster symptoms as (1) disturbance, (2) avoidance, (3) negative changes in cognition/mood, and (4) exaggerated irritability (28). 

PTSD is known as a mental health disorder resulted from psychological traumas. The disease accompanies with significant disability imposing high costs on the individual and society (29, 30). Firefighters who often face harsh environments (eg, high altitudes, darkness, and extreme heat) usually feel overwhelmed and mentally distressed (31). 

Our meta-analysis included 274 Iranian firefighters who were randomly selected and participated in 3 cross sectional studies (Table 1). In fact, various studies have asserted that younger firefighters are at higher risk of PTSD (19, 20). According to the results of the present study, the firefighters’ mean age was 35.21 years, which indicates that they had low work experience. Age was inversely correlated with PTSD, with higher incidence of this condition in older individuals and vice versa; this finding agreed with that of Godarzi et al’s study (32). This may be due to the fact that older staff with more working experience can do better in keeping their working spirit up and preserving their coping skills. In addition, the rate of harmful events declines with age which can partly explain this observation. This fact can root in capacity of older staff to perform their duties with improved coping strategies. The results of meta-regression analysis showed that the prevalence of PTSD among Iranian firefighting personnel was on the rise (Figure 3). Coincidentally, Iran has recently experienced a high incidence of natural disasters such as floods, earthquakes, and fires (such as Plasco building fire in Tehran in 2018). Firefighters are at the front line of exposure to these traumatic events, which correlates with the increasing trend of PTSD in these individuals.

Furthermore, stresses associated with driving risks, timely arrival at the scene, timely response to wireless contacts, inhaling toxic gases, physical injuries, facing gruesome scenes, round the clock shifts, fear of performing suboptimal operations, exposure to extreme heat and its side effects, and finally being at risk of explosion at the scene can predispose firefighters to PTSD. 

The prevalence of PTSD among firefighting personnel was 23.17% (Figure 2). A study by Alghamdi et al reported that the prevalence of PTSD among firefighters was 57% (14). Another study by Tomaka et al found that the prevalence of PTSD among firefighters was 32.4%, which was consistent with our study (33). Other international studies by Kim JE et al (8), Berninger et al (34) and Psarros et al (19) have reported the prevalence of PTSD in firefighters as 5.4%, 15.5%, and 18.6%, respectively, which were all lower than the rate observed in our study. In addition to different measurement tools applied, these differences may also be explained by variabilities in personal attributes, working experience, the number of distressing missions, exposure to traumatic events as well as the severity of such events. In a study by Noor et al, on firefighters in the United States, the prevalence of PTSD in male firefighters was 20% (11). A high level of social support may play an important role in preventing the PTSD of firefighters (35). Firefighters are at the forefront of a variety of disasters, such as floods, earthquakes, and fires, and are exposed to various types of stressors, which increase their risk of PTSD.

In a study by Yvon Motreff et al (31) in Paris, the prevalence of PTSD among firefighters was reported as 9.5%. They reported that the incidence of PTSD was associated with low levels of education, presence in insecure crime scenes, and lack of PTSD-related educations. Educational courses on the potential consequences of mental diseases and psychological interventions in disastrous traumatic events should be provided for firefighters.

## Limitation

The number of studies investigating the prevalence of PTSD among Iranian firefighters was limited. In the present study, the heterogeneity among the studies was high, which may be affected by different sample sizes and tools. 

## Conclusion

The aim of this study was to investigate the prevalence of PTSD among Iranian firefighters, which was obtained as 23.17%, representing a relatively high. The prevalence of PTSD has been on a rise in recent years. Considering the destructive physical and mental effects of PTSD and the roles of firefighters in managing accidents and rescuing human lives and property, appropriate functional educations about their emotional self-efficacy and coping skills should be considered to diagnose the disorder and prevent its consequent cognitive and functional effects in these individuals. In addition, organizational and social supports and psychological interventions can help to promote firefighters’ mental health. Moreover, more studies should be conducted to explore various effects of PTSD on firefighters’ mental health and life style and quality. Also, the effectiveness of preventive and therapeutic modalities in managing PTSD should be investigated in this population to prevent mental overload and the negative effects of dealing with accidents.
